# New Insights into Monocyte-Derived Macrophages in Glioblastoma

**DOI:** 10.34133/research.0836

**Published:** 2025-08-12

**Authors:** Xuetong Li, Wei Gao, Xinmiao Long, Minghua Wu

**Affiliations:** ^1^ The Affiliated Cancer Hospital of Xiangya School of Medicine, Central South University/Hunan Cancer Hospital, Changsha, China.; ^2^ The Key Laboratory of Carcinogenesis of the Chinese Ministry of Health, The Key Laboratory of Carcinogenesis and Cancer Invasion of the Chinese Ministry of Education, Cancer Research Institute, School of Basic Medicine, Central South University, Changsha, China.; ^3^ Xiangya School of Public Health, Central South University, Changsha, China.

## Abstract

Glioblastoma (GBM) is a highly aggressive brain tumor characterized by an immunosuppressive microenvironment that importantly contributes to treatment resistance. Monocyte-derived macrophages (MDMs), which comprise approximately 50% of the cellular population within the GBM microenvironment, represent a major subset of tumor-associated macrophages. These cells drive tumor progression by promoting angiogenesis, immune evasion, and the phenotypic transformation of tumor cells. MDM infiltration is mediated by specific signaling pathways and regulated by the disruption of the blood–brain barrier and tumor-associated hypoxia. Recent technological advances have uncovered substantial heterogeneity among macrophages, including hypoxia-induced, lipid-metabolizing, phagocytic, and interferon-activated subtypes. This functional diversity is shaped by tumor-specific genetic alterations and metabolic reprogramming. Therapeutic approaches focusing on MDMs include inhibiting their recruitment, enhancing phagocytic activity, employing genetically engineered macrophage, and modulating metabolic pathways. While preclinical studies suggest that these approaches may improve efficacy when combined with immune checkpoint inhibitors, the dynamic spatiotemporal heterogeneity and adaptability of macrophages within the tumor microenvironment remain substantial therapeutic challenges. Future development in combination therapies, integrating single-cell multi-omics, spatial metabolic profiling, and targeted interventions, will be critical to precisely modulate MDMs, overcome immune tolerance, and improve patient outcomes.

## Introduction

GBM is the most common and aggressive type of primary brain tumor, with an incidence rate of approximately 3.19 to 4.03 cases per 100,000 individuals. Standard treatment typically begins with maximal safe surgical resection, followed by concurrent chemoradiotherapy with the alkylating agent temozolomide (TMZ) and radiation therapy. Immunotherapeutic agents are increasingly being integrated into the standard of care to improve efficacy; for example, anti-programmed cell death-1 (PD-1) antibodies in combination with TMZ and radiotherapy have shown promising outcomes in clinical trials [1]. In addition, tumor-treating electric field therapy has demonstrated improved progression-free and overall survival in some patients. Despite these advances, GBM remains associated with high mortality. The median overall survival ranges from 14.6 to 20.5 months, and the 5-year survival rate is less than 5% to 7%.

The prognosis of GBM is influenced by a combination of patient-specific characteristics, tumor biology, and therapeutic strategies (Table 1). Among these, the immunosuppressive tumor microenvironment (TME) of GBM notably impairs the efficacy of T cell-targeted therapies. A diverse array of immunosuppressive cells, including tumor-associated macrophages (TAMs) and myeloid-derived suppressor cells (MDSCs), collectively suppress antitumor immune responses. TAMs account for approximately 50% of the viable cellular population within the GBM tumor.

**Table 1.** Key factors influencing GBM prognosis. The table summarizes critical factors influencing patient outcomes, including intratumor heterogeneity, genetic alterations, demographic variables (e.g., age), anatomical constraints (tumor location), and mechanisms of therapeutic resistance. Each factor is linked to its biological or clinical mechanism of action and supported by representative references.

Key factor	Description	Mechanism of impact	References
Intratumor heterogeneity	Spatial and molecular diversity within the same tumor	Spatial heterogeneity: low-oxygen region and normal-oxygen region; proliferative region and static region; genotype difference.	[129]
Genetic mutations	Mutations in genes such as EGFR, PTEN, LCTL, and IDH1	Drive tumor cell proliferation, invasion, and resistance to therapy	[130,131]
Patient age	Association between patient age and GBM prognosis	Younger patients tend to have better outcomes compared to older patients	[132]
Tumor location	Anatomical location of the tumor within the brain	Affects surgical resectability and risk of neurological deficits	[133]
Treatment resistance	GBM cells develop resistance to conventional therapies (radiotherapy, chemotherapy, and targeted agents)	Cellular adaptation: Tumor cells adapt to hypoxia, stress, and nutrient scarcity. Immune evasion: MDMs and immune cells in the microenvironment suppress antitumor immunity.	[8]

In recent years, combination immunotherapies targeting TAMs have received growing interest. In healthy brain tissue, microglia serve as the resident immune cells of the central nervous system (CNS), maintaining homeostasis through roles such as preserving blood–brain barrier (BBB) integrity, facilitating cerebral blood flow and cerebrospinal fluid drainage, transporting nutrients, and clearing debris and apoptotic cells [2]. However, in GBM, this homeostasis is severely disrupted. TAMs not only outnumber other immune cells but also interact with malignant cells, thereby promoting tumor progression [3]. Within the GBM microenvironment, TAMs include both MDMs and microglia [4]. According to Park et al. [5], homeostatic differentiation refers to the adaptation of monocytes to the tissue environment, allowing them to exert beneficial functions under normal physiological conditions. In contrast, allostatic differentiation occurs when disease-related signals override steady-state signals, leading to disruptions in the monocyte–macrophage differentiation process. During glioma development, this immune homeostasis in the CNS is disturbed. In this context, microglia are reprogrammed and undergo immunosuppressive allostatic differentiation into MDM-like cells [2], which are primarily located at the tumor margins [6]. Simultaneously, circulating monocytes originating from the bone marrow migrate out of the bloodstream and, through the action of various inflammatory cytokines and chemokines, cross the BBB and infiltrate the brain parenchyma, where they differentiate into MDMs. In glioblastoma (GBM), MDMs exhibit a relatively stable phenotype and are predominantly localized within the tumor core and perivascular regions, with higher prevalence in recurrent GBM [7]. MDMs not only lose their normal physiological functions, but also become important drivers of tumor progression, promoting tumor growth, angiogenesis, and immune escape, thereby aggravating the malignancy of tumors. According to Andersen et al., MDMs in GBM can be subdivided into distinct clusters, including metabolism-associated, immune-associated, and hypoxia-associated subtypes. These subsets exhibit context-specific functions and recruitment patters, reflecting the dynamic heterogeneity of the GBM microenvironment [8]. Despite increasing efforts to therapeutically target MDMs through modifying MDM recruitment and survival, enhancing phagocytic activity, or introducing gene-edited macrophages, clinical outcomes have remained limited. This is likely due to the extensive cellular diversity and plasticity of the MDMs, both within and between tumors [9].

In this article, we systematically articulate the developmental origins of MDMs, while elucidating the molecular mechanisms governing their infiltration and activation within the GBM TME. MDMs orchestrate tumor progression via angiogenesis, T-cell immunosuppression, and extracellular matrix (ECM) remodeling, exhibiting functional synergy with MDSCs. Single-cell RNA sequencing (scRNA-seq) and spatial profiling reveal context-dependent MDM heterogeneity, underscoring their plasticity in dynamically sculpting the immunosuppressive TME. Targeting MDM subsets (e.g., colony stimulating factor 1 R [CSF1R] inhibition and metabolic reprogramming) combined with immune checkpoint blockade or adoptive therapies represents a transformative strategy to overcome GBM immunotherapy resistance.

## MDM Infiltration and Activation

### MDM mobilization

Monocytes originate in the bone marrow, where hematopoietic stem cells give rise to myeloid progenitor cells, which further differentiate into monocyte progenitors and ultimately mature monocytes. Monocytes exist as distinct subpopulations with lineage-specific markers in both humans and mice. Comparative gene expression analyses have identified both conserved and species-specific features that distinguish human and murine monocyte subsets. In humans, 3 primary monocyte subpopulations have been widely recognized: (a) classical monocytes (CD14^++^CD16^−^), (b) intermediate monocytes (CD14^+^CD16^+^), and (c) nonclassical monocytes (CD14^+^CD16^++^). While this classification is broadly accepted, the biological functions of intermediate monocytes remain poorly understood. It is also unclear whether they represent a distinct lineage or a transitional state between classical and nonclassical monocytes [10]. In mouse models, monocytes are generally categorized into 2 major subsets: (a) inflammatory monocytes, which exhibit high levels of lymphocyte antigen 6, and CC chemokine receptor 2 (Ly6C^+^ CCR2^+^), which are similar to classical monocytes in humans; (b) patrol/circulating monocytes, which display lack of Ly6C and lack CCR2 (Ly6C^−^ CCR2^−^), which is the same as nonclassical monocytes in humans. Given this interspecies diversity, it is important to examine monocyte behavior in both physiological and pathological conditions [11].

Recent technological advances have substantially deepened our understanding of monocyte heterogeneity. Mass cytometry has enabled the use of surface markers such as human leukocyte antigen – DR isotype (HLA-DR), CCR2, and CD36 to characterize CD14^+^CD16^+^ intermediate monocytes [12]. Similarly, Ly6C, CD43, and Treml4 (triggering receptor expressed on myeloid cells like 4) have been utilized to identify murine monocyte subsets, especially in tissues where Ly6C^hi^ monocytes down-regulate Ly6C but maintain low expression of CD43 and Treml4. Notably, the high expression of Treml4 serves as a distinguishing feature between Ly6C^hi^ and Ly6C^lo^ subpopulations [13]. scRNA-seq analysis using Smart-Seq2 identified 2 novel human monocyte subpopulations, Mono3 and Mono4. The Mono3 subpopulation highly expresses genes related to cell cycle, differentiation, and trafficking such as MXD1, CXCR1, CXCR2, and VNN2, while the Mono4 subpopulation highly expresses genes related to cell cycle, differentiation, and transport, such as PRF1, GNLY, and CTSW; they are toxicity-related genes. [14] These findings highlight the heterogeneity within monocyte populations and underscore the need for further research to validate these subsets, develop isolation methods, and elucidate their functions.

Building upon this progress in monocyte classification, a key question emerges: how are monocytes transformed into MDMs? The first step involves monocyte egress from the bone marrow. This process is regulated by monocyte chemotactic proteins, which include CCL2, CCL7, CCL8, and MCP-4/5 (designated as CCL13 in humans and CCL12 in mice). In GBM, MCP-1 and MCP3, in particular, mobilize monocytes for activity by attracting them through activation of their CCR2 receptor. The CCR2/CCL2 axis represents one of the critical mechanisms through which MDMs are recruited in GBM and represents a key target in immunotherapeutic strategies [15]. Ly6C^hi^CCR2^−^ patrolling monocytes—often referred to as “bloodstream macrophages” because they circulate in the bloodstream and are at a differentiated stage—are active in the vasculature, checking the luminal surfaces of endothelial tubes for damage and work in tandem with neutrophils to accomplish repair [16]. Ly6C^hi^CCR2^hi^ monocytes rapidly accumulate at sites of GBM inflammation and damage. During this process, Ly6C^hi^CCR2^hi^ monocytes localize to sites of tissue defects and inflammatory damage, where they extravasate to generate MDMs and have been demonstrated to be a primary source of MDMs in GBM. MDMs from bone marrow then break through the blood vessels and BBB and enter the GBM TME. Why, then, do MDMs predominantly infiltrate GBM rather than neutrophils? The preferential infiltration of GBM by monocytes rather than neutrophils remains an intriguing phenomenon, influenced by multiple factors, including tumor location and genetic alterations. Oncogenic drivers and tumor suppressors regulate the recruitment of myeloid cells by modulating the expression of chemokines and cytokines. For example, while oncogenic RAS is rarely mutated in GBM, it is frequently activated through downstream signaling pathways and has been shown to promote neutrophil recruitment in other cancer types. In GBM models, tumors with neurofibromin 1 (NF1) silencing display increased neutrophil presence, whereas EGFR-vIII and PDGFB-driven tumors exhibit enhanced monocyte infiltration. Notably, the mesenchymal subtype of GBM—characterized by NF1 alterations—is associated with elevated neutrophil recruitment. These findings underscore the intricate relationship between genetic mutations and immune cell recruitment in GBM and suggest that the TME is shaped by both oncogenic signaling and the unique immunological features of the brain [11].

### MDMs in perivascular infiltration

The recruitment of circulating monocytes to sites of tissue damage in GBM involves traversal of the vascular endothelium and BBB. This multistep process is mediated by interactions between MDMs and vascular endothelial cells (ECs), involving initial capture, lateral migration, firm adhesion, intravascular movement, and eventual transendothelial migration [17]. MDMs stimulate BBB ECs to adopt a pro-angiogenic gene expression profile, up-regulating molecules such as vascular cell adhesion molecule 1 (VCAM1), intercellular adhesion molecule 1 (ICAM1), C-X-C motif chemokine ligand 5 (CXCL5), and CXCL10. Up-regulation of VCAM1 and ICAM1 facilitates monocyte adhesion to the endothelium and their migration into the brain parenchyma, contributing to a local pro-inflammatory milieu [18]. In contrast, CXCL5 and CXCL10, key chemokines in GBM, promote abnormal angiogenesis through endothelial activations, and their elevated expression levels are associated with poor prognosis [19].

Delprat et al. have shown that pericytes and perivascular cancer-associated fibroblasts (CAFs) play pivotal roles in recruiting MDMs to the perivascular niche. GBM cells secrete platelet-derived growth factor (PDGF)-BB, which induces interleukin-33 (IL-33) expression and secretion in pericytes and CAFs via a PDGF receptor β (PDGFRβ)-dependent mechanism. IL-33 then stimulates macrophage migration through the IL-33 receptor (ST2) pathway, thereby promoting MDM infiltration [17]. In GBM, newly recruited monocytes are differentiated into MDMs and localize to the perivascular niche through a mechanism driven by PDGF-BB secreted by GBM cells, which involves a PDGFRβ-dependent pathway that induces IL-33 expression and secretion by pericytes and CAFs, which then stimulates MDM migration in an ST2 receptor-dependent manner [20]. It has been shown that among these monocytes localized in the perivascular area, CX3CR1^lo^CCR2^hi^ monocytes are highly motile [21], and they rapidly transferred to stationary CX3CR2^hi^CCR2^lo^ and CX3CR2^lo^CCR2-MDMs in pericytes [22]. This process reveals this localization of monocytes from the circulation to damaged vessels. The transformation and localization of MDMs demonstrate that this highly kinematic state of MDMs suggests that we should consider heterogeneity and dynamic diversity when analyzing MDMs. scRNA-seq and cell indexing of transcriptomes and epitopes by sequencing (CITE-seq) have revealed important immune cell diversity, with clear distinctions in MDM abundance and phenotype between primary and recurrent GBMs. These findings reflect the evolving nature of GBM immune microenvironment and highlight the necessity of targeting MDMs as part of therapeutic strategies, particularly in recurrent GBM.

### MDM activation in BBB

As previously described, MDMs are recruited to the CNS in response to GBM-induced inflammation, primarily via a CCR2/CCL2-mediated mechanism. This recruitment is facilitated by the disruption of the BBB, a highly specialized and tightly regulated structure composed of astrocytes, ECs, and pericytes [23]. In GBM, BBB integrity is compromised not only due to inflammation and structural aberrations but also through increased angiogenesis. The aberrant angiogenesis, largely driven by elevated levels of vascular endothelial growth factor (VEGF), leads to increased vascular permeability and vessel wall damage [24]. Atrophic and dysfunctional vessels promote tumor progression and the infiltration of immune cells. Interestingly, VEGF paradoxically inhibits immune cell extravasation by down-regulating ICAM1 and VCAM1 expression [25]. As the BBB becomes more permeable, immune cells—predominantly MDMs—gain access to the brain. These MDMs congregate in hypoxic zones within the necrotic tumor core, where hypoxia further drives their polarization toward immunosuppressive phenotypes [26]. IL-33 expression is positively correlated with increased MDM infiltration, potentially through modulation of microglial chemokine secretion. However, the mechanism by which microglia and MDMs interact in this context remains poorly understood and requires further investigation [27]. Within the TME, MDMs adapt to the CNS milieu and become functionally activated. They exhibit RNA expression signatures enriched for anti-inflammatory and immunosuppressive genes, including arginase 1 (ARG1), CD39, transforming growth factor-β (TGF-β), IL-10, IL-1 receptor antagonist (IL1RN), CD155, macrophage scavenger receptor 1 (MSR1), and PD-L1 [28]. In addition, these MDMs express high levels of major histocompatibility complex (MHC) class II molecules and proangiogenic factors such as VEGF-A, reflecting both their immune-modulating and pro-tumorigenic roles.

**Fig. 1.**
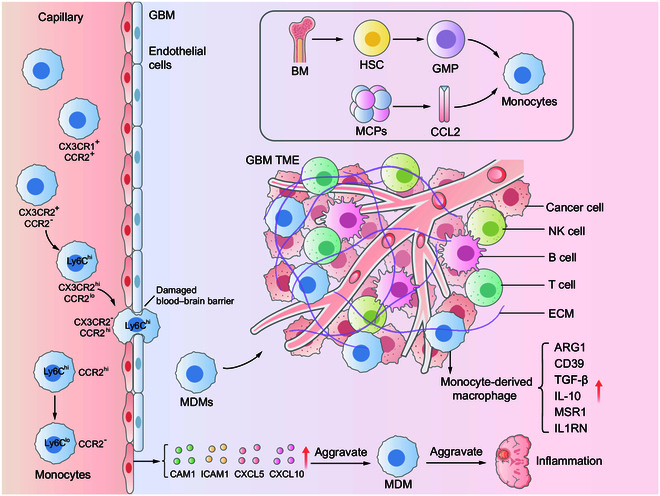
The origin of monocyte-derived macrophages (MDMs) and their entry into brain tissue. MCPs (CCL2) mediate monocyte mobilization from the bone marrow to the circulatory system via the CCR2 signaling axis. Circulating monocytes entering the bloodstream can be divided into inflammatory monocytes (Ly6C^+^CCR2^+^) and patrol monocytes (Ly6C^-^CCR2^−^). Inflammatory monocytes (Ly6C^+^CCR2^+^) localize at the site of tissue defects transforming into CX3CR2^lo^ and CCR2-MDMs. MDMs interacted with endothelial cells to penetrate the vasculature, and MDMs induced elevation of vascular cell adhesion molecule 1 (VCAM1), intercellular adhesion molecule 1 (ICAM1), C-X-C motif chemokine ligand 5 (CXCL5), and C-X-C motif chemokine ligand 10 (CXCL10), which promoted MDMs recruitment and inflammatory response. MDMs entered the microenvironment of GBM in a state of sustained activation, in which arginase 1 (ARG1), CD39, transforming growth factor-β (TGF-β), interleukin-10 (IL-10), interleukin-1 receptor antagonist (IL1RN), and macrophage scavenging receptor 1 (MSR1) were elevated.

These findings indicate that GBM not only orchestrates the spatiotemporal infiltration of MDMs into the TME but also dictates their functional polarization. We aim to elucidate the molecular mechanisms that govern MDM-mediated immunosuppression, including interactions with tumor-derived cytokines, metabolic reprogramming, and epigenetic modifications—processes that collectively suppress antitumor immunity (Fig. 1).

## Functions of MDMs

### Acceleration of abnormal vascularization

MDMs are the predominant immune cell population infiltrating GBM and play a central role in disease progression. Rapid tumor cell proliferation creates a severely hypoxic microenvironment and depletes nutrients, conditions that promote abnormal angiogenesis and support the development of an immunosuppressive TME [29]. Under hypoxic conditions, MDMs secrete various chemokines and angiogenic factors, most notably VEGF-A, which contributes to immunosuppressive vascularization. Cui et al. demonstrated that M2-like MDMs produce high levels of TGF-β1 and IL-10, both of which facilitate endothelial proliferation and angiogenesis through the integrin αvβ3 and Src–PI3K–YAP signaling pathways. Coinhibition of integrin αvβ3 and TGF-β1 suppresses neovascularization mediated by TAM–EC interactions [30]. Additionally, cat eye syndrome critical region protein 1 (CECR1) expression is up-regulated in MDMs and modulates vascular remodeling via the PDGFB–PDGFRβ signaling axis. This signaling enhances communications between MDMs and vascular wall cells, promoting pericyte activation and angiogenesis [31].

During tumor progression, abnormal vascular structures not only support GBM growth but also enhance interactions between MDMs and tumor cells, thereby aggravating tumor invasiveness. For example, MDMs are densely distributed in preinvasive regions of GBM, where they induce CD133^+^ glioma stem-like cells via the secretion of TGF-β1, contributing to increased tumor aggressiveness [32]. Additionally, pleiotrophin (PTN) secreted by CD163^+^ MDMs binds to protein tyrosine phosphatase receptor type Z1 (PTPRZ1) on the surface of glioblastoma stem cells (GSCs). This interaction between PTN and PTPRZ1 is crucial for maintaining the role of CSCs, thereby promoting the rapid growth of GBM [33]. MDMs also release IL-6 and IL-1β, which respectively activate phosphoglycerate kinase 1 via the 3-phosphoinositide-dependent protein kinase 1 pathway and glycerol-3-phosphate dehydrogenase through the PI3K/PKCδ pathway in GBM cells [34]. These metabolic activations enhance glycolysis and proliferation in GBM cells. Overall, MDMs exhibit potent proangiogenic and tumor-supportive properties in GBM, suggesting that they represent a promising therapeutic target. A critical component of the GBM TME is its immunosuppressive nature. The following section explores how MDMs contribute to this immunosuppression.

### Contribution to immunosuppression

MDMs contribute to multiple immunosuppressive mechanisms that impair tumor-specific T-cell responses in GBM [35]. A central pathway involves activation of the aryl hydrocarbon receptor (AHR) by tumor-derived metabolites such as kynurenine, produced via indoleamine 2,3-dioxygenase 1 (IDO1), tryptophan 2,3-dioxygenase, or IL-4I1 [36]. AHR activation increases MDM recruitment and up-regulates immunosuppressive genes. The inducible transcription factor KLF4 facilitates MDM recruitment, while cytokine signaling suppressor SOCS2 mediates an immunosuppressive microenvironment by dampening nuclear factor κB (NF-κB) signaling and promoting the expression of exonuclease CD39, which works with CD73 to generate adenosine, an inhibitor of T-cell activity. This leads to both increased recruitment of MDMs and reduced T-cell immunocompetence. In addition, FOXP3^−^ regulatory T cells have also been shown to support AHR activation [37] and further restrict effector T-cell function [38]. Immune checkpoint molecules also contribute to immunosuppression. GBM cells accelerate monocytosis in an autocrine and paracrine manner via IL-10 signaling and induce PD-L1 expression on MDMs. In vitro, IL-10-activated MDMs induce T-cell apoptosis, a process that can be reversed by IL-10 inhibition [39]. Additionally, Yao et al. showed that CD133^+^CSCs promote B7-H4 expression on MDMs via the IL-6/JAK/STAT3 (signal transducer and activator of transcription 3) axis. Interactions involving B7-H4 and MDMs are linked to poor GBM prognosis [40].

Monocyte-derived MDSCs (M-MDSCs) in humans are characterized by low or absent MHC class II and HLA-DR expression. Another subset, polymorphonuclear or granulocytic MDSCs (PMN-MDSCs), represents over 80% of the total MDSC in most cancers [41]. A third subset, early-stage MDSCs (e-MDSCs), is found almost exclusively in GBM [42] and is categorized as immature due to the absence of definitive differentiation markers [43]. Although e-MDSCs show the weakest ability to inhibit T-cell proliferation, their accumulation appears to have no marked impact on patient survival [44]. MDSC markers vary prominently. In mice, CD11b and Gr1 are used to identify MDSCs, with Ly6G and Ly6C distinguishing PMN-MDSCs (Ly6G^+^/Ly6C^lo^/CD11b^+^) from M-MDSCs (Ly6G^−^/Ly6C^hi^/CD11b^+^) [45]. CD49d is recognized as a specific marker for M-MDSCs, while lectin-like oxidized low-density lipoprotein receptor 1 (LOX1) has emerged as a specific marker for PMN-MDSCs [46]. In humans, PMN-MDSCs are identified by the markers CD14^−^/CD11b^+^/CD66b^+^/CD15^+^, and M-MDSCs are defined by HLA^−^/DR^−/low^/CD11b^+^/CD15^−^/CD14^+^ [47]. In the context of GBM, vascular noninflammatory molecule 2 (VNN2^+^) has been proposed as a unique marker for MDSCs [48].

In the process of research on MDSCs, various mechanisms of exerting immunosuppressive functions of MDSCs have been summarized; for example, in the TME, the metabolism of the MDSC population undergoes reprogramming, showing enhancement of lipid metabolism [49], glucose metabolism, such as glycolytic processes, and the pentose phosphate pathway [50]; in addition, amino acids, as an important component in the microenvironment, particularly arginine and tryptophan in the MDSC population, and cysteine metabolism levels were also conspicuously elevated in the MDSC population [51]. MDSCs impair CD8^+^ T-cell function through the secretion of immunosuppressive molecules such as ARG1, IL-10, and TGF-β [52]. For example, a glioma-associated MDSC subset induced by granulocyte-macrophage colony-stimulating factor (GM-CSF) and expressing IL-4 receptor alpha has been shown to suppress T cells via ARG1. Given this diversity in cellular phenotype and function, it is critical to investigate the heterogeneous states of MDSCs and elucidate the therapeutically relevant mechanisms that drive the activity of specific subpopulations [53] (Table 2).

**Table 2.** Difference between MDSCs and MDMs

	Origin	Subtype	Marker/Signature	Function	Reference
MDSC	CMP monocytic precursors	M-MDSC	HLADR^−/low^/CD11b^+^/CD15^−^/CD14^+^ (Human) Ly6G^−^/Ly6C^hi^/CD11b^+^ (Mouse) CD49d (novel markers)	Suppresses the anti-tumor response of T cells through multiple mechanisms. For example: Exert an inhibitory effect on the immunocompetence of CD8^+^ T cells through molecules such as arginase 1 (ARG1), interleukin-10 (IL-10), and transforming growth factor-β (TGF-β)	[36–38]
		PMN-MDSC	CD14^−^/CD11b^+^/CD66b^+^/CD15^+^ (Human) Ly6G^+^/Ly6C^lo^/CD11b^+^ (Mouse) LOX1 (novel markers)		[39]
		e-MDSC	CD123, CD14, CD15, CD16		[40,41]
MDMs	Myeloid progenitor cells	Hypoxia-MDMs	P50, HIF-1α, C-Jun	Multiple heterogeneous groups of MDMs play different roles in GBM. For example: Accelerate abnormal vascularization	[57]
		Lipid-MDMs	Dysregulation of lipid metabolism		[59]
	Monocyte progenitors	Phagocytic-MDMs	Performs a variety of phagocytic roles	Contribute to immunosuppression	[65]
		IFN-γ-induced MDMs	CXCL9, CXCL10, IFN-γ	Promotes phenotype transformation	[71]

CMP, common myeloid progenitor, MDSC, myeloid-derived suppressor cell; M-MDSC, monocytic MDSC; PMN, polymorphonuclear; e-MDSC, early-MDSC; CXCL9, C-X-C motif chemokine ligand 9; CXCL10, C-X-C motif chemokine ligand 10

### Promotion of phenotypic transformation

MDMs also play crucial roles in promoting the phenotypic transformation of GBM tumor cells and promoting the interaction between tumor cells and other types of cells, such as ECs, among others. MDMs could promote the transformation of GBM cells into a mesenchymal (MES)-like state. In addition, in terms of GBM metabolism, lactate, a key metabolite produced by GBM cells, modulates MDM polarization and C-X-C matrix chemokine receptor 4 (CXCR4) signaling also promotes mesenchymal–epithelial transition within GBMs, resulting in shorter survival. Additionally, Oncostatin M (OSM), secreted by MDMs, activates STAT3 signaling through its receptor OSMR or leukemia inhibitory factor receptor subunit alpha and GP130, promoting MES-like subtype transitions in GBM cells [54]. MDMs are often found adjacent to GBM-associated ECs, where they participate not only in aberrant angiogenesis but also in VEGF-mediated MDM polarization [55], representing another axis of phenotypic transformation. Immune checkpoints may further drive tumor-supportive phenotypes. For instance, Tim-3, a well-established coinhibitory receptor in GBM, promotes tumor cell proliferation and supports macrophage polarization toward a malignant phenotype via the IL-6 signaling pathway. DExH-box helicase 9 (DHX9), a potent RNA repressor, enhances MDM infiltration and polarization toward an M2-like phenotype. Inhibition of DHX9 reduces the effect of CSF-1 and enhances the inhibitory effect of transcription factor 12 (TCF12) [56]. The expression of VCAM1 and ICAM1, driven by bradykinin receptor 1 (B1R) and IL-1β, enhances GBM cell motility. B1R also stimulates the secretion of pro-oncogenic factors, including CCL5, IL-6, CXCL11, and IL-8, which facilitate the MDM infiltration into the TME [57]. This cytokine and chemokine network contributes to MDM heterogeneity and phenotypes, and is implicated in treatment resistance.

## MDM Diversity

MDMs are dynamic in the GBM microenvironment, varying not only in spatial dimensions but also in heterogeneous groups over time. The development of scRNA-seq and spatial genomics has led to a deeper understanding of the heterogeneity of MDMs, and here we focus on the spatiotemporal heterogeneity of MDMs and its impact on tumor progression [50]. First, in terms of temporal changes, the heterogeneity of MDM population was obviously different in different periods and stages of GBM tumor progression [58]. For example, in the early stage of GBM tumors, MDMs mainly exhibit the M1 subtype, which mainly exhibits an oncogenic effect. With the progression of the tumor and the change of invasiveness, MDMs gradually begin to shift to the M2 type, at which time the population of MDMs exhibits the function of promoting tumor progression. In previous studies, some representative subgroups of MDMs have been shown to exist in certain specific spatial locations. For example, hypoxia-MDMs have been reported to be located primarily in hypoxic zones at the margins of necrotic regions of tumors, and are limited by the size of the hypoxic space [59]. Another study has also revealed a distinctly hierarchical structure of cellular composition in GBM. One population of inflammation-associated MDMs or immune-associated MDMs with other malignant tumor cells also has a distinct hierarchical structure, and the dynamics of this structure also appears to be hypoxia-driven [60]. All these findings demonstrate the important role of hypoxia, a central factor, in the spatial distribution of MDMs in GBM. The current studies have analyzed a large number of MDMs using CD45, CD11b, or lineage tracing techniques to isolate defined cell populations in mouse models to better understand the heterogeneity of MDMs. Several markers were identified in these studies to distinguish microglia from MDMs. More MDM subpopulations have been identified with the technology out of single-cell histology, and each subtype has been categorized based on its specific gene expression and functional characteristics. Based on a large body of literature and authors’ investigations and classifications, we summarize the 4 most important and distinguishable subpopulations based on a synthesis of various subpopulation types and subpopulation characteristics. These include hypoxia-MDMs, lipid-MDMs, phagocytic-MDMs and IFN-γ-induced MDMs. Next, we will introduce the specific functions and specific immune mechanisms of each subpopulation of MDMs in the hope of providing directions and foundations for future research.

### Hypoxia-MDMs

Hypoxia is a hallmark of GBM, and hypoxia-associated MDMs (hypoxia-MDMs) have emerged as key contributors to GBM progression and therapy resistance [61]. These MDMs are actively recruited to hypoxic regions of the TME, where they adopt conserved transcriptional signatures across both human and murine models. Their polarization is primarily driven by a p50-mediated NF-κB signaling pathway and is shaped by hypoxic tumor cues that promote angiogenesis and neovascularization. Hypoxia-MDMs contribute to tumor progression by enhancing vascular elasticity, facilitating the migration of mesenchymal-like tumor cells, modulating T-cell activity and immunosuppression, and promoting ECM degradation. Collectively, properties underscore hypoxic stress as a central driver of GBM malignancy. Meanwhile, hypoxia-activated mesenchymal stromal cells contribute to tumor progression by destabilizing endothelial junctions through the secretion of adrenomedullin, leading to the formation of hyperpermeable vasculature [62]. In the TME of GBM, hypoxia-MDMs represent a unique population of MDMs that are enriched in hypoxic regions of GBMs and are influenced by hypoxic tumor cues that promote neovascularization. Previous studies have elucidated key features of hypoxia-MDMs in GBMs, including conserved transcriptional profiles in human and mouse GBM tissues and cell models. P50-mediated hypoxic response programs drive their polarization, and these cells is crucial in enhancing tumor vascular elasticity, promoting migration of mesenchymal-like cells, influencing T-cell activation and increasing ECM degradation role. Together, these findings suggest that hypoxic stress is an important driver of GBM progression. In addition, hypoxia-MSCs destabilize endothelial junctions via adrenomedullin (ADM), which leads to the formation of hyperpermeable tumor vasculature, whereas VE-adhesin adhesion junctions are critical for restoring vascular integrity [59].

**Fig. 2.**
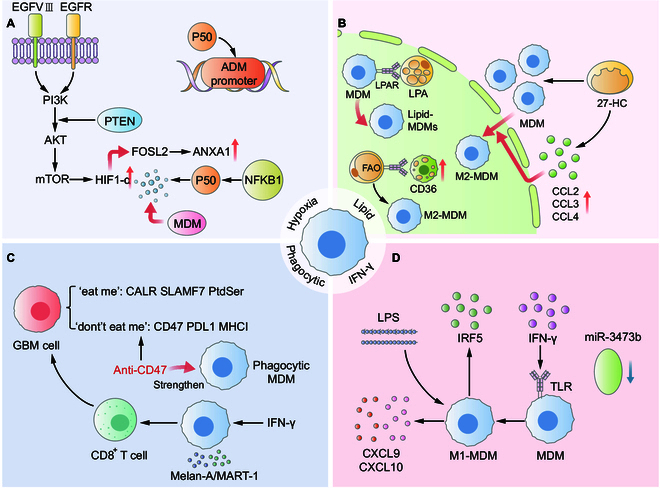
Classification of MDMs in GBM. (A) Hypoxic MDMs: EGFR mutations or PTEN deletion activates the PI3K/AKT/mTOR pathway, up-regulating HIF-1α. NF-κB and P50 sustain hypoxic-MDM growth, while the HIF-1α–FOSL2 axis promotes tumor invasion and elevates ANXA1, maintaining MDM phenotype. P50 up-regulation also increases ADM, disrupting endothelial cell connectivity. (B) Lipid-MDMs: Enhanced fatty acid oxidation (FAO) drives MDM conversion to M2-MDM, with high CD36 expression in tumor cells. Lysophosphatidic acid (LPA) binding to LPAR and elevated 27-HC promote chemokine release (CCL2/3/4), recruiting MDMs and sustaining lipid metabolism. (C) Phagocytic-MDMs: Phagocytosis is mediated by CALR, SLAMF7, and Ptdser, while CD47, PD-L1, and MHC1 inhibit it. CD47 blockade enhances phagocytosis. (D) IFN-γ-activated phagocytic-MDMs present MART-1 antigen to T cells, triggering T cell activation and tumor cell phagocytosis. Interferon-γ-type MDMs: Down-regulation of miR-3473b and TLR binding promote M1-MDM transformation, releasing CXCL9/10. LPS-induced IRF5 release maintains M1-MDM status.

Beyond angiogenesis, hypoxia also alters MDM polarization. Transcriptomic analysis using gene set enrichment analysis has shown that NF-κB signaling is highly up-regulated in hypoxia-MDMs, with p50, HIF-1α [63], and c-Jun serving as key transcription factors regulating hypoxic MDMs. Genetic alterations in GBM—such as EGFR amplification or mutation and PTEN deletion—further stabilize HIF-1α through the PI3K/AKT/mTOR pathway, amplifying the hypoxic response. Other modulators, including FAT1, integrins αvβ3 and αvβ5, HIG2, and GGTI, also regulate HIF-1α expression, contributing to increased tumor cell migration and invasion. Notably, p50 knockdown importantly reduces the expression of hypoxia-MDM signature genes, reinforcing its critical role in hypoxia-MDM signaling [64]. Furthermore, scRNA-seq studies have identified FOSL2 as a highly expressed gene in aged GBM lesions. Activation of the HIF-1α–FOSL2 axis under hypoxia drives tumor invasiveness. FOSL2 also regulates the expression of ANXA1, which facilitates the differentiation of monocytes into hypoxia-adapted, immunosuppressive MDMs. These cells suppress CD8^+^ T-cell activity, presenting a significant barrier to effective immunotherapeutic strategies [65] (Fig. 2A).

### Lipid-MDMs

Tumor cells exhibit rapid proliferation and consume large amounts of nutrients, resulting in a hypoxic, acidic, and nutrient-depleted TME. In response, MDMs undergo extensive reprogramming—particularly of lipid metabolism—to sustain their function. This reprogramming involves enhanced fatty acid uptake, fatty acid oxidation (FAO), and activation of related pathways that generate an alternative energy source. These changes give rise to a lipid-associated MDM phenotype (lipid-MDM). A hallmark of lipid-MDMs is the extracellular leakage of fatty acids, which contributes to the formation of a lipid-rich microenvironment [66]. In response, lipid-MDMs increase fatty acid uptake and accumulate intracellular lipid droplets. Simultaneously, FAO is up-regulated, while glycolysis is suppressed. This metabolic reprogramming promotes polarization of lipid-MDMs toward the M2-like immunosuppressive phenotype, reinforcing their protumorigenic roles [67]. One key regulator of this phenotype is CD36, a scavenger receptor that mediates fatty acid uptake [68]. CD36 expression has been strongly associated with the polarization of lipid-MDMs. Recent studies by Luo et al. demonstrated that lipid-MDMs exhibit reduced phagocytic activity and elevated PD-L1 expression, contributing to T-cell suppression and enhancing their M2 immunosuppressive phenotype [69].

In addition to the CD36 molecule mentioned above, phospholipid content also influences the phenotype of lipid-MDMs. For example, lysophosphatidic acid (LPA) and phosphatidylserine (PS) in GBM are associated with patient prognosis. LPA binds to the LPA receptor (LPAR) on the surface of monocytes, which then activates PPAR-γ through activation of the PI3K/AKT/mTOR signaling pathway, which leads these monocytes into lipid-MDMs and their polarization to an M2 phenotype [70]. In addition, 27-hydroxycholesterol (27-HC), a cholesterol derivative, has been shown to accelerate GBM progression. Elevated 27-HC promotes the differentiation of MDMs and induces the secretion of CCL family chemokines, which further recruit monocytes. Collectively, these findings suggest that the development of lipid-MDMs is largely driven by cholesterol efflux and utilization [66] (Fig. 2B).

### Phagocytic-MDMs

Phagocytosis, the engulfment and degradation of extracellular material such as pathogens or apoptotic cells, is a fundamental function of macrophages. In the CNS, MDMs play essential roles in neurodevelopment, homeostasis, and the response to pathological insults, including acute brain injury and various neurological disorders [71].

In GBM, phagocytic activity is regulated by the interplay of “eat-me” and “don’t-eat-me” signals expressed on tumor cells. “Eat-me” ligands (e.g., calreticulin, SLAMF7, regulatory antibodies, and PS) promote phagocytosis through cytoskeletal rearrangement in MDMs. Conversely, “don’t-eat-me” signals (e.g., CD47, PD-L1, and MHC I) bind to signal-regulatory protein α (SIRPα) on macrophages, inhibiting myosin II conformational changes and thus blocking phagosome formation [72]. CD47, in particular, is a potent inhibitor of phagocytosis in GBM [73]. Therapeutic strategies targeting this axis have shown promise. Anti-CD47 or anti-SIRPα can restore phagocytic capacity, enhance antigen presentation, and promote CD8^+^ T-cell responses. In vitro and in vivo models have demonstrated that anti-CD47 treatment enhances phagocytosis by murine macrophages and boosts presentation of tumor-derived antigens via MHC I to OT-I T cells [74]. These experimental results were also confirmed in GBM tumor cell experiments in mice [75]. Chung et al. [76] found that T-cell activation can also be activated by IFN-γ. Taken together, due to the cross-presentation capability of macrophages, they may play a pivotal role in enhancing immune cell responses, particularly under certain conditions or in response to targeted therapies [71] (Fig. 2C).

### IFN-γ-induced MDMs

IFN-γ, also known as a “macrophage-activating factor”, is a central mediator of macrophage activation and a driver of M1-like polarization. IFN-γ primes MDMs for Toll-like receptor (TLR)-mediated responses and shifts them toward a pro-inflammatory, antitumor M1 state by down-regulating miR-3473b, a microRNA that promotes M2-like polarization [77]. Additionally, IFN-γ suppresses sterol regulatory element-binding protein 1 (SREBP1)-dependent fatty acid synthesis in M2-like MDMs [78]. M1-like macrophages are considered more cytotoxic and are crucial for tumor immunosurveillance [79]. Upon IFN-γ stimulation, MDMs produce chemokines such as CXCL9 and CXCL10, which promote immune cell infiltration into the TME and may also inhibit angiogenesis [80].

MDMs constitute a heterogeneous population influenced by both oxygen availability (hypoxic vs. normoxic) and tumor progression stage (early vs. advanced) [81]. During early tumorigenesis, M1-type macrophages are typically recruited and respond to inflammatory cues by releasing pro-inflammatory cytokines. These signals facilitate the differentiation of T helper 1 (Th1), Th17, and natural killer (NK) cells. M1 macrophage activation is commonly triggered by bacterial components and lipopolysaccharide (LPS); however, cytokines such as GM-CSF and IFN-γ, secreted by MDMs, also contribute to autocrine activation and M1 polarization. Moreover, interferon regulatory factor 5 supports the persistence of the M1 phenotype by enhancing lymphocyte proliferation, Th1/Th17 responses, and the transcription of IL-12p35 and IL-12p40 [82] (Fig. 2D). These findings emphasize the immunostimulatory potential of IFN-γ-primed MDMs and their therapeutic relevance to reverse immune suppression within GBM TME.

## Strategies for Targeting MDMs

### Modulating MDM recruitment and phagocytic activity

The CCR2/CCL2 signaling axis plays a central role in the recruitment of monocytes—including MDMs and MDSCs—into the TME, where it exerts pro-angiogenic and pro-inflammatory effects. The recruitment is primarily mediated by CCL2, a chemokine secreted by cancer cells, which binds to CCR2 on circulating monocytes. CCR2 inhibition enhances the efficacy of immune checkpoint inhibitors [83]. However, its therapeutic benefit in patients with GBM remains uncertain, with limited clinical success reported so far [84]. A possible reason for the limited efficacy of CCL2/CCR2 inhibition is the involvement of additional chemokines in MDM recruitment within GBM. For instance, angiogenic inducers such as VEGF-A and CSF1 also contribute to monocyte recruitment and subsequent differentiation into MDMs [85]. Furthermore, cytokines such as CCL5 and CCL4 [86] have been implicated in facilitating MDM infiltration into the GBM microenvironment [58]. This suggests that monotherapy targeting the CCR2/CCL2 axis may be insufficient and that combinatorial approaches involving other chemokine inhibitors may yield better outcomes. For example, carlumab, a neutralizing antibody against CCL2, showed modest antitumor effects in patients [87] with prostate cancer. One possible explanation for the ineffective therapeutic outcomes observed in GBM is that the inhibitory response to the CCL2/CCR2 axis varies across different immune cells. In GBM, CCR2 knockout mouse models revealed an inverse correlation between macrophage-identified MDM and microglia-identified MDM, suggesting that the antitumor effects of CCR2 blockade may be more closely associated with modulating microglial responses than MDM inhibition. Furthermore, other signaling pathways modulating the CCL2/CCR2 axis have also been identified. For instance, kynurenine, a metabolite secreted by GBM cells, activates AHR in MDMs, leading to up-regulation of CCR2 and enhanced MDM infiltration. This pathway can be counteracted using AHR antagonists such as CH-223191 [88]. Other chemokine–receptor pairs have also been implicated in MDM recruitment and function in GBM, including osteopontin (OPN)/αvβ5 integrin [89], lysyl oxidase (LOX)/β1 integrin, and slit guidance ligand 2 (SLIT2)/ROBO1/2 [90]. Therapeutic strategies focusing on disrupting these interactions using 4-1BB–OPN bispecific aptamer, neutralizing antibodies, or SLIT2-capture protein Robo1Fc have shown promise [91].

Moreover, circadian regulator CLOCK (circadian locomotor output cycles protein kaput) has emerged as a novel player in MDM infiltration. Overexpression of CLOCK promotes massive MDM infiltration by up-regulating olfactory ligand-like 3 (OLFML3) and leguminous protease (LGMN). Therefore, treatment can be achieved by inhibiting CLOCK with an axis between the transcriptional target of OLFML3 and LGMN [92]. Disruption of the CLOCK-OLFML3/LGMN axis impairs GBM tumor growth and MDM infiltration. However, OLFML3 and LGMN receptors on MDM need to be further investigated [93]. Another combinatorial approach is using the CCR2 inhibitor CCX872 combined with anti-PD-1 therapy, which importantly increased survival, reduced MDSC infiltration, and enhanced T-cell cytotoxicity in vivo [94].

**Fig. 3.**
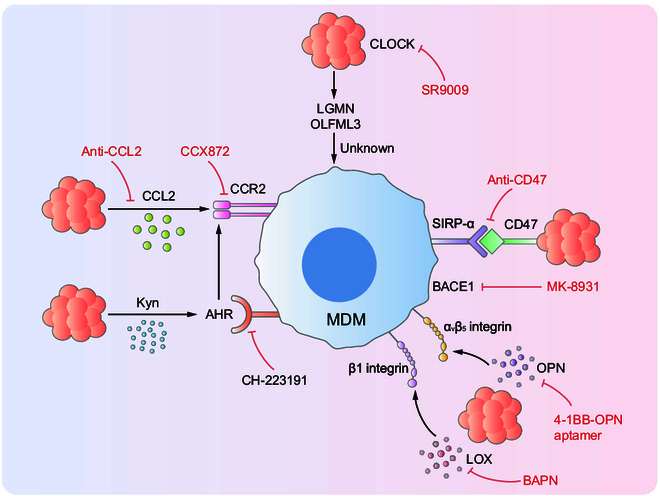
Targeting recruitment and phagocytosis of MDMs. Kyn, kynurenine; AHR, aryl hydrocarbon receptor; LGMN, asparagine peptidase; OLFML3, olfactory mediator-like 3; CLOCK, circadian locomotor output cycles protein kaput; SIRP-α, signal-regulated protein α; BACE1, β-amyloid precursor protein cleavage enzyme 1; OPN, osteoblastin; LOX, lipoxygenase.

In addition to their immunomodulatory activities, MDMs retain the ability to phagocytose tumor cells [95]. However, many GBM cells overexpress CD47, a “don’t eat me” signal that binds to SIRPα on MDMs, thereby suppressing phagocytosis. Depletion of CD47 has been shown to restore macrophage-mediated phagocytosis of tumor cells and suppress GBM growth [95], highlighting the therapeutic potential of targeting the CD47/SIRPα axis [96,97]. Other studies have further reported that treatment with human anti-CD47 antibodies resulted in more favorable outcomes in pediatric glioma than in adult GBM [98]. Although CD47 inhibition has demonstrated potential against GBM, complete tumor remission remains limited. Several factors may play a role, including variable epitope accessibility due to antibody molecular size and pharmacokinetics [99], high intratumoral heterogeneity, and insufficient pro-phagocytic costimulatory signals within the TME. To overcome these limitations, combination strategies have been explored. In murine GBM models, anti-CD47 antibodies combined with TMZ and anti-PD-1 therapy [98]. Furthermore, the addition of etomoxir, a carnitine palmitoyl transferase 1 inhibitor, improves efficacy [100]. Additionally, the development of a herpesvirus-encoded lysosomal agent has been reported to enhance BBB penetration and reduce infusion-associated toxicity, while exhibiting strong cytotoxic effects against GBM cells [101]. Targeting SIRPα directly is another promising strategy, as studies have demonstrated that therapeutic nanobodies can be effectively delivered to tumors in murine models [102]. In addition to targeting the CD47/SIRPα axis, inhibition of BACE1 by drugs promoted MDM-mediated phagocytosis of GSCs (inhibitor MK-8931), which also affected the progression of GBM in vivo (Fig. 3). Nevertheless, combination therapy approaches must address critical challenges such as potential drug–drug interactions, off-target toxicities, and hematological side effects. Moreover, the efficiency of drug delivery systems and the precision of targeting mechanisms require further refinement. Collectively, these findings suggest that enhancing MDM-mediated phagocytosis represents a promising, though still developing, strategy for the treatment of GBM [84].

### Reversing immunosuppressive reprogramming of MDMs

Disruption of colony-stimulating factor 1 (CSF1) signaling via its receptor (CSF1R) can deplete specific subsets of macrophages [103]. Consequently, CSF1R inhibitors have been developed and investigated for their potential in GBM treatment [104]. However, despite achieving effective MDM depletion and adequate tumor drug penetration in pharmacodynamic studies, clinical trials have failed to show marked clinical improvements [105].

**Fig. 4.**
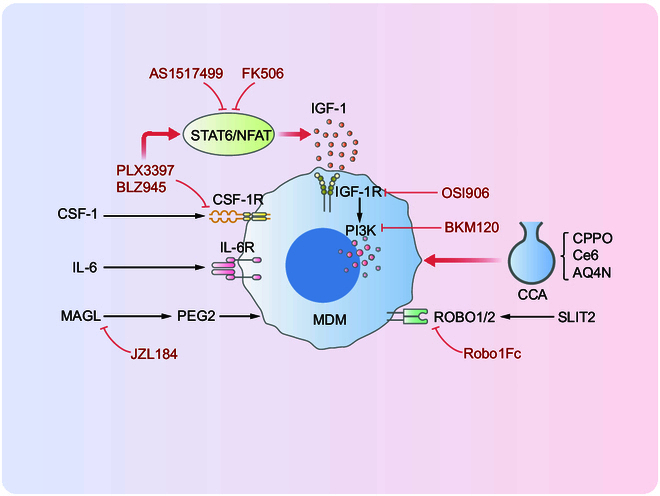
Immunosuppressive effects targeting MDMs. CSF-1, colony-stimulating factor 1; CSF-1R, CSF-1 receptor; MAGL, monoacylglycerol lipase; PGE2, prostaglandin E2; IGF-1, insulin-like growth factor 1; SLIT2, slit guidance ligand 2; ROBO1/2, roundabout receptor 1/2.

CSF1R inhibitors such as PLX3397 and BLZ945 not only deplete macrophages but also interfere with their polarization [106]. For instance, BLZ945 treatment in murine GBM models did not reduce MDM infiltration but blocked their pro-tumorigenic polarization. While CSF1R inhibition initially suppresses tumor progression, prolonged treatment has been linked to adaptive resistance in tumor cells [107]. Mechanistically, CSF1R inhibitors activate the STAT6/NFAT signaling pathway in MDMs, triggering the secretion of insulin-like growth factor 1 (IGF-1). IGF-1 stimulates the IGF-1R/PI3K pathway in GBM cells, enhancing their invasiveness. Cotreatment with the IGF-1R inhibitor OSI906 or the PI3K inhibitor BKM120 can counteract this effect. Additionally, the STAT6 inhibitor AS1517499 and the NFAT-calcineurin inhibitor FK506 can suppress STAT6/NFAT signaling in MDMs, enhancing the antitumor effects of BLZ945 [107]. BLZ945 also demonstrated the potential to synergize with anti-PD-1 therapies in preclinical models [108]. Despite promising preclinical findings, PLX3397 has failed to demonstrate clinical benefits in patients with GBM. Current clinical trials are evaluating combinatorial strategies, including CSF1R inhibitors with IGF-1R/PI3K inhibitors, radiotherapy, or immune checkpoint blockade. The limited improvement in survival may be partly due to the unique anatomical and microenvironmental features of GBM. Although CSF1R inhibitors can cross the BBB and reduce MDM at the tumor sites, they may inadequately penetrate the tumor margins and surrounding normal brain tissue, leaving these regions susceptible to recurrence. Future efforts should prioritize improved drug delivery to tumor margins and peritumoral tissues [109]. Strengthening the efficacy evaluation of CSF1R inhibitors such as PLX3397 and BLZ945 remains a substantial challenge for GBM therapy development [110]. Moreover, CSF1R inhibition has been associated with fibrosis within the CNS, which can create a protective niche for tumor cells. Fibrotic environment may prolong tumor cell survival, promote recurrence, exacerbate inflammation, and delay tissue repair, ultimately contributing to poor clinical outcomes [107]. Another major barrier to the successful use of CSF1R inhibitors is the development of drug resistance. While the mechanisms of tumor cell resistance are well understood, it remains unclear whether CSF1R inhibition alters the overall composition and dynamics of the GBM TME, and whether similar effects are observed in other types of brain tumors. Alternative strategies to reverse MDM immunosuppression have also been proposed. These include the capture protein Robo1Fc, P-selectin inhibitor KF38789, 4-1BB–OPN conjugate, and BACE1 inhibitor MK-8931 [111–113]. Combination therapies involving Robo1Fc with anti-PD-1 or anti-4-1BB–OPN agents have demonstrated enhanced antitumor efficacy. However, key questions remain for clinical translation, including whether these combinations can effectively reduce MDM infiltration, prevent tumor recurrence, and minimize drug-related side effects while improving durability of response. Moreover, minimizing drug-related side effects and improving the durability of therapeutic efficacy represent important areas for future clinical research. In addition, a drug was developed consisting of extracellular vesicles of immunostimulated macrophages loaded with the chemo excitation source CPPO (C), the photosensitizers Ce6 (C), and the hydrophilic hypoxia-activated prodrug AQ4N (A) (collectively referred to as CCA). It has been demonstrated in experimental models in mice that CCA has potent efficacy in reprogramming MDMs and inhibiting GBM tumor development [113]. Since MDMs are immunosuppressive, their reprogramming may enhance the efficacy of immune checkpoint inhibitors (ICIs). Indeed, in preclinical GBM models, targeted MDM reprogramming by IL-6 inhibition with CD40 stimulation [114], SLIT2 inhibition, and monoacylglycerol lipase inhibition showed strong synergistic effects with ICIs such as anti-PD-1, anti-CTLA4, and anti-4-1BB. In conclusion, these findings suggest that the role of microenvironmental reprogramming targeting MDMs is a very innovative strategy that, on the one hand, suppresses tumor aggressiveness and also improves the efficacy of existing immunotherapies against GBM (Fig. 4).

### Engineering chimeric antigen receptor–macrophage therapies

Chimeric antigen receptor (CAR) cell therapies, such as CAR-T and CAR-NK cells, have shown promise in various cancers but involve complex procedures, including cell isolation, genetic modification, and ex vivo expansion prior to patient infusion. In contrast, CAR–macrophage (CAR-M) therapy is emerging as a powerful alternative for GBM due to its potential. Chen et al. developed an intracavitary injectable hydrogel–nanocarrier system that enables in situ generation of CAR-M cells within the tumor resection cavity. This strategy delivers CAR genes directly to the surgical site, thereby minimizing systemic side effects while maintaining therapeutic efficacy within the TME. CAR-M cells specifically target and phagocytose CD133^+^ glioma stem cells (GSCs), eliminating residual tumor-initiating cells and inducing a robust adaptive immune response. This localized approach prevents postoperative GBM recurrence by establishing sustained antitumor immunity. In GBM mouse models, CAR-M therapy substantially reduced tumor burden, prolonged survival, and improved immune activation. Furthermore, combination therapy with anti-CD47 antibodies enhanced phagocytosis, suppressed immunosuppressive cells, and improved tumor cell clearance. These findings underscore the therapeutic potential of CAR-Ms in postoperative GBM settings. However, clinical validations in humans are required to assess efficacy, safety, and the potential combinatorial approaches with other immunotherapeutic drugs [115].

To enhance CAR-M functionality, Lei et al. engineered second-generation CAR-M cells incorporating a dual-signaling structure composed of the CD3ζ domain and the Toll/IL-1 receptor (TIR) domain of TLR4. The CD3ζ domain boosts effector functions traditionally associated with T cells, while the TIR domain activates NF-κB signaling, promoting M1-like macrophage polarization. Compared to first-generation CAR-Ms, this dual-signaling configuration allows sustained antitumor polarization within the immunosuppressive GBM TME. These CAR-M cells showed enhanced phagocytic activity, effective tumor recognition, and superior antitumor efficacy. The incorporation of the TIR domain not only amplified phagocytosis but also triggered innate and adaptive immune responses, fostering long-term immune memory and improving the durability of treatment outcomes.

**Fig. 5.**
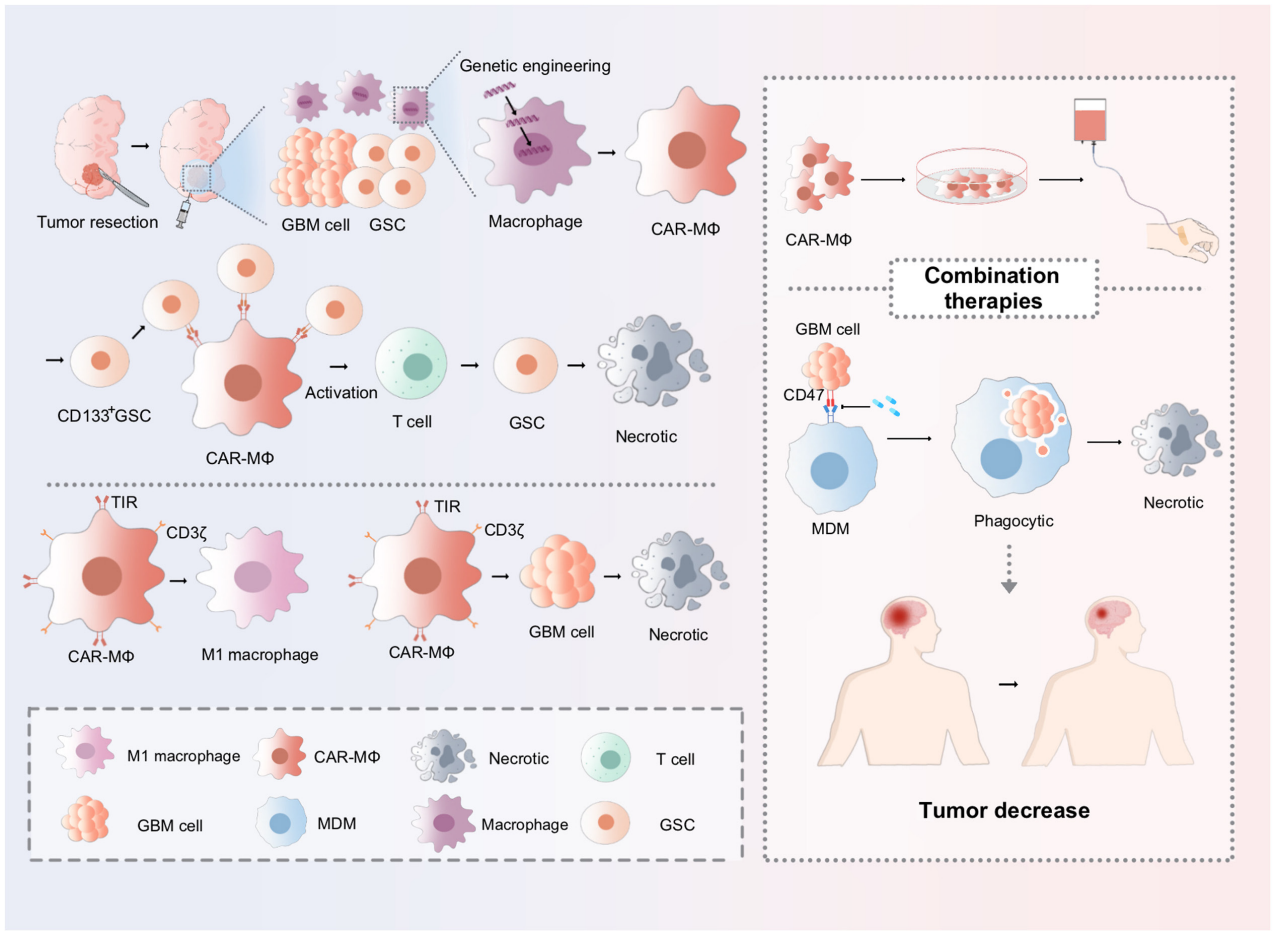
Current CAR-M therapies based on injectable hydrogel-nanocarrier systems and second-generation CAR-M therapies in combination with anti-CD47 drugs; CAR-M cells are generated locally in the tumor resection cavity to avoid systemic toxicity. CAR-M achieves precise phagocytosis by recognizing CD133^+^ glioma stem cells (GSCs) and removes residual tumors through activation of the adaptive immune response. Combined with anti-CD47 antibody to block “don’t eat me” signaling (CD47/SIRPα axis), importantly enhancing macrophage clearance of GSCs. Fusion of a T cell effector domain (CD3ζ) with an innate immune activation domain (TLR4-TIR) drives M1-type polarization through the NF-κB pathway.

Lei et al. also investigated the synergistic potential of combining second-generation CAR-M cells and anti-CD47 antibodies. CD47, often overexpressed on tumor cells, serves as a “don’t eat me” signal by engaging SIRPα on macrophages to suppress phagocytosis. Blocking the CD47–SIRPα axis with monoclonal antibodies facilitates macrophage activation and tumor clearance. Dual therapy with second-generation CAR-M cells and anti-CD47 antibodies produced importantly greater antitumor effects compared to monotherapies alone [116]. While these preclinical and early-phase clinical findings are encouraging, further investigation is essential to optimize the safety, efficacy, and scalability of CAR-M therapies in GBM. Integrating CAR-M therapy with other immunotherapeutic approaches remains a complex challenge. For example, while CAR-M therapy enhances macrophage phagocytosis, it may also affect the survival and function of other immune cells within the TME. Moreover, potential exhaustion of MDMs and the long-term sustainability of treatment efficacy must be carefully considered. Systemic administration of anti-CD47 agents may lead to widespread macrophage depletion, which could impair the physiological roles of tissue-resident macrophages in host defense and immune surveillance. This systemic exhaustion may result in adverse outcomes and negatively affect clinical prognosis. Therefore, strategies to minimize off-target effects and preserve immune homeostasis are critical for the clinical success of CAR-M-based therapies (Fig. 5).

### Targeting MDM metabolism for therapeutic intervention

Metabolic reprogramming plays an important role in both GBM progression and immune suppression within the TME. In tumor and immune cells alike, changes in metabolism greatly influence tumor progression and invasion [117]. Consequently, targeting specific metabolic pathways has emerged as a promising strategy. One important metabolic enzyme is IDO1, a rate-limiting enzyme that converts tryptophan to kynurenine. IDO1 is expressed by GBM cells and has been shown to suppress T-cell responses via multiple mechanisms, including activation of the AHR in MDMs [118]. In GBM mouse models, IDO1 expression in nonmalignant cells also promoted tumor progression, suggesting that the combined use of IDO1 inhibitors with radiotherapy and immunotherapy can improve treatment efficacy [119]. However, the efficacy of IDO1 inhibition may be limited by the activity of IL-4I1, a tryptophan catabolic enzyme that drives AHR activation in gliomas and other malignancies. This metabolic redundancy may explain that targeting IDO1 alone may be insufficient for fully blocking AHR-mediated immunosuppression in GBM, and that a broader spectrum of AHR antagonists is needed.

In addition to tryptophan metabolism, arginine metabolism also plays a central role in immune regulation within the GBM TME. Elevated expression of ARG1 and ARG2 has been observed in both rat and human GBM. These enzymes catabolize L-arginine, an amino acid essential for the proliferation of T cells and NK cells. Increased arginase activity depletes L-arginine availability, thereby suppressing antitumor immunity. Transcriptomic and single-cell analyses have shown that ARG1 and ARG2 are expressed not only in GBM cells but also in tumor-infiltrating MDMs. Inhibiting ARG1/2 can restore L-arginine availability, thereby enhancing antitumor immune responses. The arginase inhibitor OAT-1746 has shown notable efficacy in blocking MDM-mediated tumor invasiveness, crossed the BBB, and elevated brain L-arginine levels in mice bearing GL261 gliomas. Importantly, oral administration of OAT-1746 maintained cell viability and did not alter the immune cell composition of the TME, as confirmed by transcriptomic analysis. Its mechanism involves modifying gene expression in CD11b^+^ immune cells, contributing to a more conducive TME. When used in combination with anti-PD-1 checkpoint blockade, OAT-1746 significantly reduced glioma burden and enhanced overall antitumor efficacy. These findings support the hypothesis that ARG1/2 inhibition in tumor cells and MDMs can enhance the immune response by restoring metabolic conditions favorable for T and NK cell activity, thereby increasing the efficacy of immunotherapeutic agents such as PD-1 inhibitors.

The metabolic heterogeneity of GBM presents a significant barrier to therapy. Recent advances in radiomics and metabolomics have enabled more precise profiling of metabolism-associated genes. These tools also hold promise for prognostic assessment and therapy personalization based on metabolic profiling [120].

## Conclusion

It is well established that the M1/M2 classification of macrophages has been extensively used over the years. This classification is based on the differential response of macrophages to various cytokines and stimuli [121]. M1 macrophages are typically pro-inflammatory macrophages, whereas M2 macrophages are anti-inflammatory immunoregulatory macrophages. IFN-γ, LPS, and tumor necrosis factor-alpha (TNF-α) enhance Th1 responses and activate NK cells through the induction of pro-inflammatory cytokines [122]. In contrast, M2-type MDMs are primarily activated by peroxisome proliferator-activated receptor gamma and STAT6 [123]. The secretion of anti-inflammatory cytokines, such as IL-6, IL-10, and IL-13, can promote cancer stemness and contribute to the development of therapeutic resistance.

During the progression of GBM, the polarization of M1- and M2-type MDMs is dynamic [124]. In the early stages, MDMs predominantly exhibit an M1-like phenotype, characterized by tumor cell killing and enhancement of T and NK cells, thereby inhibiting tumor growth [125]. As previously noted, monocytes recruited via the CCL2/CCR2 axis are typically localized in the perivascular regions, whereas CX3CR1^+^ MDMs accumulate in the peritumoral areas [126]. However, as the TME evolves, it promotes a phenotypic switch in MDMs toward an M2-like state that supports tumor invasion and immunosuppression. Upon reaching a critical threshold, MDMs rapidly transition to the M2 phenotype, further driving GBM progression [21]. Notably, few studies have investigated the precise timing of this phenotypic transition, making it difficult to determine the stage at which the M2 phenotype becomes predominant during tumor development. Therefore, defining MDMs in GBM based solely on the M1/M2 paradigm is oversimplified. In this review, we summarized 4 distinct MDM subtypes commonly observed in GBM—hypoxia-MDMs, lipid-MDMs, phagocytic-MDMs, and IFN-γ-induced MDMs—based on prior experimental evidence and functional characterizations. The biological functions and pathological roles of these subtypes in GBM require further exploration and refinement in future studies.

Despite the availability of a wide array of immunotherapeutic agents in contemporary medicine, the primary objective is to attenuate the detrimental effects of MDMs on GBM TME. However, several critical factors must be considered when evaluating the clinical efficacy of these agents. First, although certain drugs can successfully penetrate the BBB and attain high concentrations within the tumor foci, others, particularly small-molecule inhibitors, require adjunctive materials or advanced delivery systems to reach the brain parenchyma [127]. Second, the emergence of drug resistance remains a substantial challenge for both monotherapy and combination regimens. The inherent heterogeneity of GBM, along with the spatiotemporal variability in MDM phenotypes, may render specific therapeutic targets ineffective even at elevated drug concentrations. Third, the use of multi-agent or multi-targeted therapies demands careful optimization to enhance therapeutic efficacy while minimizing off-target effects. Achieving precise drug targeting and delivery remains a major challenge in the development of successful combination therapies [128]. Fourth, in the context of multidrug combinations, transient elevations in systemic cytokine levels may amplify inflammatory responses and promote tumor invasiveness. Therefore, the meticulous control of dosing schedules and drug interaction profiles is essential to ensure patient safety. In conclusion, further investigations are warranted to evaluate the potential of rationally designed combination regimens to enhance treatment outcomes for patients with GBM.

The immunosuppressive microenvironment of GBM is a central barrier to its therapeutic success, with MDMs playing a pivotal role in driving tumor progression through mechanisms such as angiogenesis, immune evasion, metabolic crosstalk, and phenotypic remodeling. Recent advances in single-cell sequencing and spatial transcriptomics have revealed the remarkable heterogeneity of these macrophages and their dynamic interactions with tumor cells, ECs, and other immune populations. Although preclinical strategies targeting these cells, such as blocking recruitment signals, enhancing phagocytic activity, modulating metabolism, and developing engineered macrophage therapies, show promise, their clinical translation remains limited by the plasticity of the TME, persistence of therapy-resistant macrophage subsets, and challenges in drug delivery across the BBB.

Future research should focus on resolving the spatiotemporal dynamics of MDMs’ function within the tumor by leveraging spatial multiomics and intravital imaging to map their real-time states across distinct tumor regions. A deeper understanding of the metabolic–immune axis, particularly the interplay between lipid metabolism and immunosuppressive functions, will enable the development of dual-target agents that disrupt metabolic fueling and immune escape mechanisms. Intelligent drug delivery systems such as bioinspired nanocarriers can enhance tumor-specific drug accumulation while activating local antitumor immunity. Clinically, adaptive frameworks that integrate molecular subtyping and the dynamic monitoring of treatment responses are essential for personalized therapeutic strategies. Cross-species studies using humanized models and organoid-immune cell cocultures will further elucidate the complex interactions between macrophages and tumor stem cells, thereby accelerating target validation and drug discovery.

By unraveling the biological complexity of MDMs and their role in GBM progression, precise interventions targeting their functional adaptability and metabolic dependencies have transformative potential. Integrating cross-disciplinary technologies and novel therapeutic paradigms, such as in situ vaccination combined with cellular therapies, may ultimately overcome the current therapeutic limitations and redefine outcomes for this devastating disease.
